# Gray mold and anthracnose disease detection on strawberry leaves using hyperspectral imaging

**DOI:** 10.1186/s13007-023-01123-w

**Published:** 2023-12-19

**Authors:** Baohua Zhang, Yunmeng Ou, Shuwan Yu, Yuchen Liu, Ying Liu, Wei Qiu

**Affiliations:** 1 College of Engineering, Nanjing Agricultural University, No. 40, Dianjiangtai Road, Taishan Street, Pukou District, Nanjing, Jiangsu 210031 P.R. China; 2 College of Artificial Intelligence, Nanjing Agricultural University, Nanjing, Jiangsu P.R. China

**Keywords:** Leaf diseases detection, Hyperspectral imaging, Spectral fingerprint features, Vegetation indices (VIs), Feature fusion, Machine learning

## Abstract

**Background:**

Gray mold and anthracnose are the main factors affecting strawberry quality and yield. Accurate and rapid early disease identification is of great significance to achieve precise targeted spraying to avoid large-scale spread of diseases and improve strawberry yield and quality. However, the characteristics between early disease infected and healthy leaves are very similar, making the early identification of strawberry gray mold and anthracnose still a challenge.

**Results:**

Based on hyperspectral imaging technology, this study explored the potential of combining spectral fingerprint features and vegetation indices (VIs) for early detection (24-h infected) of strawberry leaves diseases. The competitive adaptive reweighted sampling (CARS) algorithm and ReliefF algorithm were used for the extraction of spectral fingerprint features and VIs, respectively. Three machine learning models, Backpropagation Neural Network (BPNN), Support Vector Machine (SVM) and Random Forest (RF), were developed for the early identification of strawberry gray mold and anthracnose, using spectral fingerprint, VIs and their combined features as inputs respectively. The results showed that the combination of spectral fingerprint features and VIs had better recognition accuracy compared with individual features as inputs, and the accuracies of the three classifiers (BPNN, SVM and RF) were 97.78%, 94.44%, and 93.33%, respectively, which indicate that the fusion features approach proposed in this study can effectively improve the early detection performance of strawberry leaves diseases.

**Conclusions:**

This study provided an accurate, rapid, and nondestructive recognition of strawberry gray mold and anthracnose disease in early stage.

## Background

Strawberry is one of the most popular fruits with high nutritional value and economic effect. With the expansion of strawberry planting area year by year, the influence of strawberry diseases is aggravated. Gray mold and anthracnose are two of the most destructive diseases in the growth of strawberry. When the strawberry leaves appear damage or necrosis, gray mold would infect the injured part under low temperature and humidity, resulting in strawberry fruit decay. Anthrax can cause local spots on the leaves of strawberry plants. In severe cases, the whole plant will wither and die [2, 37]. These two diseases seriously affect the yield of strawberry and limit the development of strawberry food economy.

A common method for monitoring strawberry diseases is laboratory test, which includes indicator plant leaflet grafting (IPLG), electron microscopy (EM) and so on [55]. These scientific methods are more accurate than visual inspection but inefficient, time-consuming and destructive, requiring precision instruments and rigorous operation. Therefore, an accurate and non-destructive identification technology of gray mold and anthracnose in early stage is critical to strawberry production management [23, 61].

Hyperspectral imaging technology combines the advantage of imaging technology and spectral technology to obtain continuous and narrow band image information and spectral data information of each pixel [19, 24, 39, 60]. Therefore, Hyperspectral imaging, a relatively new non-destructive detection technique [16], has been proved to have a wide application prospect in the detection of plant diseases [5, 43, 50, 51, 56]. In the past few years, numerous studies used hyperspectral imaging to detect plant diseases. For instance, Nguyen et al. [38] used VIs and three-dimensional convolutional neural networks (3D CNN) to identify the grapevine vein-clearing virus (GVCV) of grapevines at the early asymptomatic stages. The reference study showed that the machine learning combined with hyperspectral imaging technology is useful to the early detection of plant disease. Chen et al. [8] obtained hyperspectral VIs for leaf spot detection by identifying sensitive bands. The reference study showed that hyperspectral imaging technology is highly feasible for detecting the occurrence of peanut leaf spot. Deng et al. [11] proposed a non-destructive citrus huanglongbing (HLB) field detection method based on hyperspectral reflectance which can identified the leaves at three different stages (healthy, symptomatic HLB-infected, and asymptomatic HLB-infected). The reference study showed that the SVM model achieved 90.8% accuracy in three-group classification which imply the hyperspectral reflectance has great potential in early plant diseases detection [25] presented an early detection model based on the partial least squares linear discriminant (PLSLD) analysis method of hyperspectral images, extracted the normalized difference TFs and VIs. The reference study showed that only by VIs and NDTIs, the classification accuracy approached 87% and 84% respectively. Meanwhile, the accuracy by using the fusion features can reach up to 90%. Guo et al. [17] fused the spectral (OWs), texture features (TFs) and VIs of hyperspectral images to establish a SVM model and applied it to the recognition of yellow rust in wheat leaves. The study presented that the fusion scheme could reach 95.8% accuracy, which was higher than only based on the OWs, VIs, and TFs (83.3%,89.5%, and 86.5% respectively). These studies imply that the fusion of different hyperspectral features is effective for early detection of plant diseases.

The above studies illustrated that hyperspectral imaging technology is feasible for detecting strawberry diseases. However, most of above diseases detection strategies only consider single feature such as spectrum, VIs and texture, which are not well adaptable to unstructured environments. And many studies proved that the method of combining diverse features to identify plants diseases is useful. In addition, the fusion feature not only can improve the accuracy of identification model, but also can improve the stability and robustness of the detection model. Consequently, the main purpose of this work was to establish a robust methodology for early detection of strawberry leaves disease based on hyperspectral imaging. The objectives were to: (1) Collect hyperspectral images of healthy and infected leaves (24-h infected) using a hyperspectral imaging system; (2) Extract spectral and VIs from the preprocessed hyperspectral images; (3) Select the spectral fingerprint and significant VIs using CARS and ReliefF algorithm, respectively; (4) Combine the spectral fingerprint and significant VIs as inputs of diverse machine learning models for early identification of strawberry leaves.

## Materials and methods

### Strawberry leaf cultivation and pathogen inoculation

Abundant strawberry leaves, which were used as objects during the experiment, are from Jiangsu Agricultural Expo Garden (China). The strawberry leaves without visual flaws (breakage, withered, spot etc.) and with good and similar nutriture were selected for experiments. Finally, a total of 360 strawberry leaves were adapted. Among them, 240 leaves were randomly selected to be infested with fungus (120 only infested with gray mold pathogens and 120 only with anthracnose) and the remainder were used as control. Anthracnose and gray mold pathogens, which were used to inoculate the strawberry leaves, are from Jiangsu Academy of Agricultural Science (China). The two different pathogens inoculum incubated on two different petri dishes were taken advantage of inoculating strawberry leaves when the mycelium bestrewed the medium. The 5 mm small round mycelium, which scattered on the edge of petri dishes, was picked out with a sterile toothpick and smeared at the surface of each leaf. Then, the diseases successfully infected the leaves. It was worth noting that at the beginning, clean water mist was used to sprinkle all samples which was vital to enhance the success rate of the diseases vaccination. Subsequently, the 240 infected (120 only infested with gray mold pathogens and 120 only with Anthracnose) and 120 uninfected samples were separately kept in different growth containers with the equal controlled environment which with temperature (20 °C) and relative humidity (90.0%) and 12 h light/dark cycle. Finally, the hyperspectral images of 240 infected (120 only infested with gray mold pathogens and 120 only with anthracnose) and 120 healthy samples were collected 24 h after inoculation.

### Hyperspectral image collection and processing

#### Hyperspectral imaging system

**Fig. 1:**
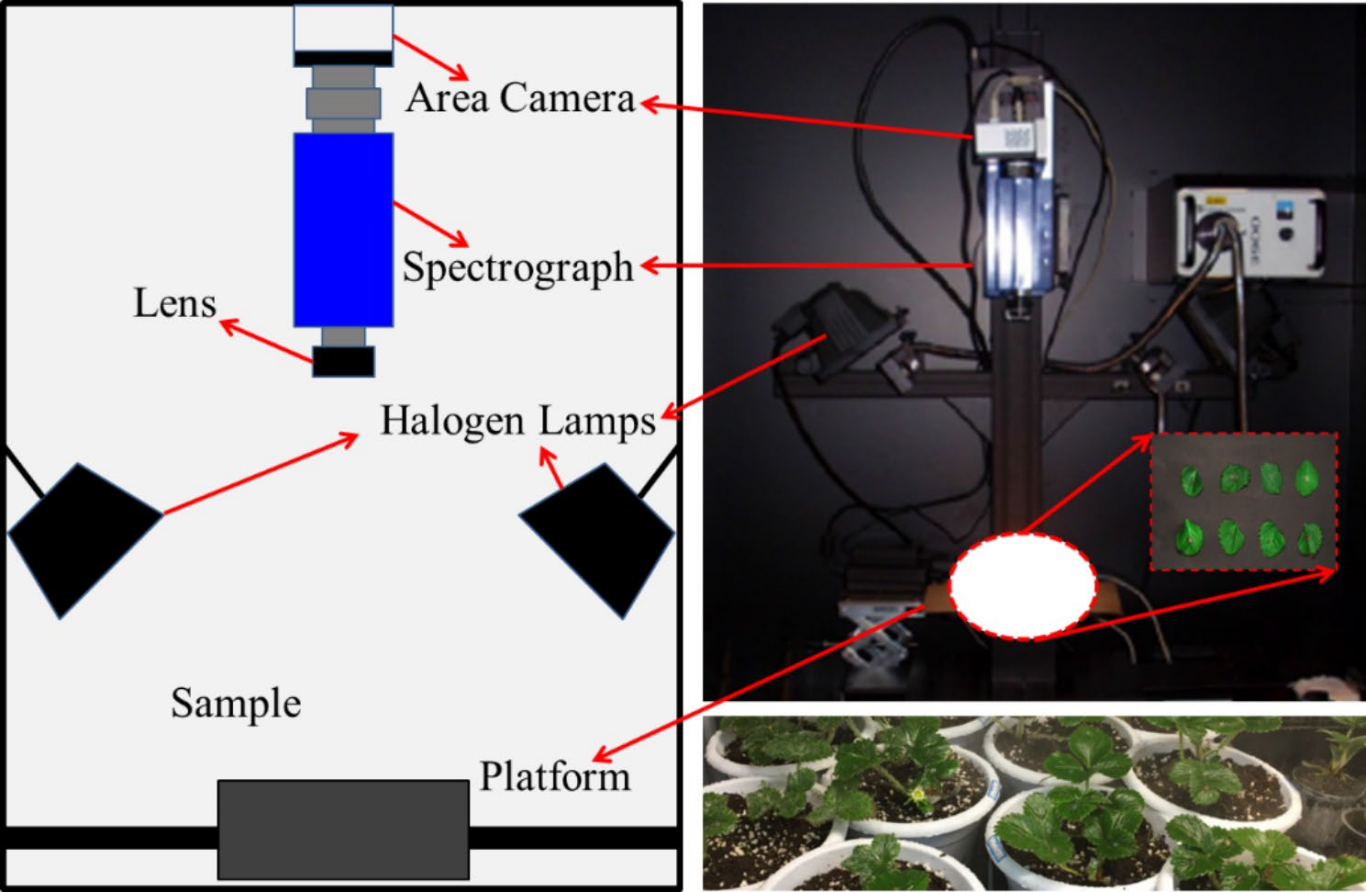
The schematic diagram of the hyperspectral imaging system

The spectral images were acquired by a hyperspectral imaging (HIS) system. The configuration of the HIS supplied in this study was presented in Fig. 1. The illuminating system contains two 150 W halogen lamps adjusted with the height of 40 cm and at angle of about 45° to illuminate the camera’s field of view. The spectral range was from 400 to 1050 nm. As for the specific hardware configuration of HIS, it can be referred to the previous article [59]. There was a software (Isuzu Optics Corp, Taiwan, China), which was provided to be compatible with the computer. It was able to set the related parameter of HSI.

#### Image acquisition and calibration

**Fig. 2:**
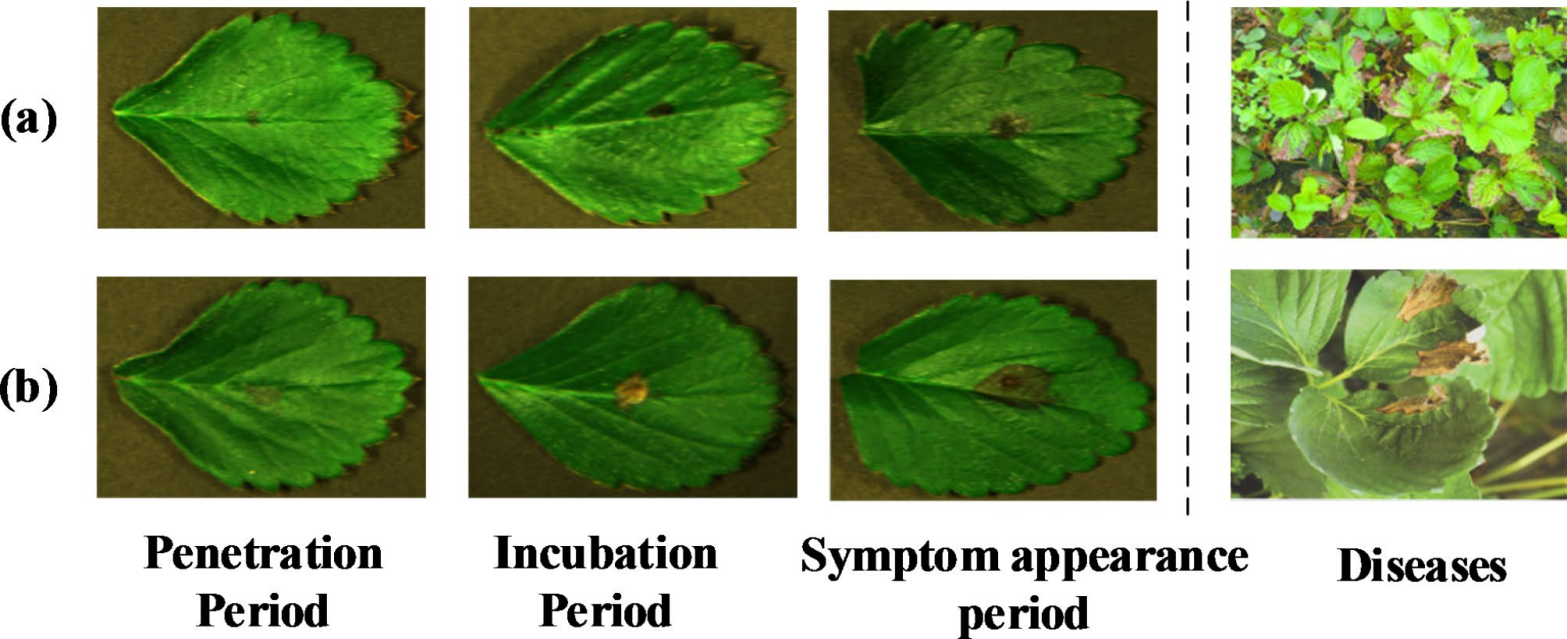
The figure of penetration, incubation, symptom and widespread appearance period. (**a**) Anthracnose. (**b**) Gray mold

In this study, the strawberry leaves were scanned line by line on a removable platform and the vertical distance between this platform and the camera is 50 cm. The speed of the mobile platform is adjusted to 0.8 mm/s and the exposure time is fixed at 26 ms, which can avert the emergence of distortion availably. Finally, 240 hyperspectral images (healthy and 24-h infected) were acquired. The hyperspectral images of deferent stage of infected samples (penetration period, incubation period, symptom appearance period and widespread period) were shown in Fig. 2. The spot in the infected leaves of 24 h is not obvious. It can be difficult to identify the diseases by the naked eye. And the outward appearances of the leaves with gray mold and anthracnose disease were similar.

The inevitable dark current in CCD left some spectral data with a great deal of noise, which generated the appearance of a large amount of bootless interference information resulting in the decrease of disease detection accuracy. Hence, the noises required to be eliminated by calibrating the original hyperspectral images before the images are used for extracting feature [62]. Therefore, the raw hyperspectral images are needed to revise by the following equation before they were used for detection.

1 $${R}_{i}=\left(\frac{{RS}_{i}-{RD}_{i}}{{RW}_{i}-{RD}_{i}}\right)\times 100\%$$

where ${R}_{i}$ and RS represents the corrected hyperspectral reflectivity image and the strength values of equal pixels of the sample image, respectively. Relevant parameters RD represents the dark reference image, which can be gained when the camera lens in the shelter of non-reflective and opaque. RW represents the white reference image and it can be gained by measuring a spectral image of the Teflon white board with a 99.9% reflectance.

Raw hyperspectral data usually contain a wealth of irrelevant information and noise, which can disturb the detection model [64]. Therefore, in order to eliminate the influence of these irrelevant information and noise, preconditioning of spectral data become necessary. In this study, Savitzky–Golay (S-G) smoothing was engaged in the preprocessing [28, 36].

### Data extraction

#### Extraction of spectral data

The changes in spectral absorption are closely related to the substance molecular structure. When the strawberry leaves are infected with gray mold and anthracnose, the internal molecular structure of the leaves will be changed markedly [66]. The spectral reflectance between healthy and infected leaves will be significantly different. Therefore, the spectrum can be used to identify plant diseases. The spectral information was extracted in the Regions of interest (ROI). In this study, a 20 × 20 pixels ROI which contained the imperceptible spot and its surroundings was manually defined from each sample. The spectral data of one sample was designated by computing the mean of all pixel spectral reflectance values. The whole procedure was executed the HSI Analyzer software (Isuzu Optics Corp., Taiwan. Finally, 360 samples containing 120 healthy and 240 infected were obtained. Afterwards, the whole samples were repeatedly and randomly divided into a training set and a testing set in a 3:1 ratio by the Kennard Stone algorithm. The training set with 180 infected (90 only infested with gray mold pathogens and 90 only with Anthracnose) and 90 healthy samples were used for training the performance of the detected model, and the testing set with 60 (30 only infested with gray mold pathogens and 30 only with anthracnose) and 30 uninfected samples were used to the extract the VIs.

#### Extraction of vegetation indices

VIs represents the physiological structure of plant including pigment content, water, cellular structure and so on [13, 48]. When the pathogens infected the strawberry leaves, the structure of strawberry leaves will be changed, such as making chlorophyll contents decline. Hence, spectral VIs were calculated to discriminate and identify crop diseases and had achieved good performance in a large amount of research [25, 35, 45]. In this study, 25 VIs related to crop diseases were selected from the articles for the detection of gray mold and anthracnose. The equations of each VIs were listed in Table 1.

**Table 1:** Vegetation indices used in this study

No.	Category	Vegetation index	Acronym	Equation	References
1	Pigment	Red-green index	RGI	$\mathop R\nolimits_{690} /\mathop R\nolimits_{550}$	[1]
2		Photochemical reflectance index	PRI	$\left( {\mathop R\nolimits_{531} - \mathop R\nolimits_{570} } \right)/\left( {\mathop R\nolimits_{531} + \mathop R\nolimits_{570} } \right)$	[31]
3		Structure insensitive vegetation index	SIPI	$\left( {\mathop R\nolimits_{800} - \mathop R\nolimits_{445} } \right)/\left( {\mathop R\nolimits_{800} + \mathop R\nolimits_{680} } \right)$	[27]
4		Nitrogen reflectance index	NRI	$\left( {\mathop R\nolimits_{570} - \mathop R\nolimits_{670} } \right)/\left( {\mathop R\nolimits_{570} + \mathop R\nolimits_{670} } \right)$	[22]
5		Normalized chlorophyll pigment ratio index	NCPI	$\left( {\mathop R\nolimits_{670} - \mathop R\nolimits_{450} } \right)/\left( {\mathop R\nolimits_{670} + \mathop R\nolimits_{450} } \right)$	[15]
6		Plant pigment ratio	PPR	$\left( {\mathop R\nolimits_{550} - \mathop R\nolimits_{450} } \right)/\left( {\mathop R\nolimits_{550} + \mathop R\nolimits_{450} } \right)$	[57]
7		Plant senescence reflectance index	PSRI	$\left( {\mathop R\nolimits_{660} - \mathop R\nolimits_{510} } \right)/\mathop R\nolimits_{760}$	[33]
8	Structure	Simple ratio	SR	$\mathop R\nolimits_{900} /\mathop R\nolimits_{680}$	[42]
9		Greenness index	GI	$\mathop R\nolimits_{554} /\mathop R\nolimits_{667}$	[14]
10		Narrow-band normalized difference	NBNDVI	$\left( {\mathop R\nolimits_{850} - \mathop R\nolimits_{680} } \right)/\left( {\mathop R\nolimits_{850} + \mathop R\nolimits_{680} } \right)$	[20]
11		Normalized difference vegetation index	NDVI	$\left( {\mathop R\nolimits_{800} - \mathop R\nolimits_{670} } \right)/\left( {\mathop R\nolimits_{800} + \mathop R\nolimits_{670} } \right)$	[21]
12		Red-edge NDVI	RNDVI	$\left( {\mathop R\nolimits_{750} - \mathop R\nolimits_{705} } \right)/\left( {\mathop R\nolimits_{750} + \mathop R\nolimits_{705} } \right)$	[47]
13		Optimized soil-adjusted vegetation index	OSAVI	$\left( {1 + 0.16} \right) \times \left( {\mathop R\nolimits_{800} - \mathop R\nolimits_{670} } \right)/\left( {\mathop R\nolimits_{800} + \mathop R\nolimits_{670} + 0.16} \right)$	[44]
14		Ratio analysis of reflection of spectral chlorophyll b	RARSb	$\mathop R\nolimits_{675} /\left( {\mathop R\nolimits_{700} \times \mathop R\nolimits_{650} } \right)$	[3]
15		Green NDVI	GNDVI	$\left( {\mathop R\nolimits_{750} - \mathop R\nolimits_{540} + \mathop R\nolimits_{570} } \right)/\left( {\mathop R\nolimits_{750} + \mathop R\nolimits_{540} - \mathop R\nolimits_{570} } \right)$	[6]
16		Modified simple ratio	MSR	$\left( {\mathop R\nolimits_{800} /\mathop R\nolimits_{670} - 1} \right)/\sqrt {\left( {\mathop R\nolimits_{800} /\mathop R\nolimits_{670} + 1} \right)}$	[34]
17		Water band index	WI	$\mathop R\nolimits_{900} /\mathop R\nolimits_{970}$	[26]
18	Physiology and Water content	Fluorescence ratio index 1	FRI1	$\mathop R\nolimits_{690} /\mathop R\nolimits_{630}$	[58]
19		Fluorescence ratio index 2	FRI2	$\mathop R\nolimits_{750} /\mathop R\nolimits_{800}$	[12]
20		Fluorescence ratio index 3	FRI3	$\mathop R\nolimits_{690} /\mathop R\nolimits_{600}$	[12]
21		Normalized pheophytization index	NPQI	$\left( {\mathop R\nolimits_{415} - \mathop R\nolimits_{435} } \right)/\left( {\mathop R\nolimits_{415} + \mathop R\nolimits_{435} } \right)$	[7]
22		Red-edge vegetation stress index	RVSI	$\left( {\mathop R\nolimits_{{714}} +\mathop R\nolimits_{{752}} } \right)/2 - {R_{733}}$	[32]
23		Fluorescence curvature index	FCI	$R_{683}^2/\left( {{R_{675}} \times {R_{691}}} \right)$	[38]
24		Red edge position	RRE	$\left( {\mathop R\nolimits_{670} - \mathop R\nolimits_{780} } \right)/2$	[10]
25		Water stress and canopy temperature	WSCT	$\left( {\mathop R\nolimits_{970} - \mathop R\nolimits_{850} } \right)/\left( {\mathop R\nolimits_{970} + \mathop R\nolimits_{850} } \right)$	[38]

### Feature selection

#### Selection of spectral fingerprint features

Plenty of wave points, collinearity of data and redundant information existed in original hyperspectral of strawberry leaves resulting in the increase of data dimension, the reduction of inspection speed and the accuracy of classification model. Hence, there are a certain degree of hardships to detecting the two different leaves diseases directly by using the original hyperspectral. Therefore, reducing the dimension of the original spectral data and selecting the spectral fingerprint features, which can enhance the differences of hyperspectral between different types of leaves and boost the accuracy of classification model, was essential to the disease detection of strawberry leaves. The common use effective variable selection methods contain CARS, genetic algorithm (GA), successive projections algorithm (SPA) and so on. Multiple researches verify that CARS algorithm acquitted itself brilliantly in eliminating redundant wavelength variables and selecting effective wavelength variables [65]. In addition, it can solve the problem of exponential explosion caused by inordinate number of spectral as well. Hence, in this study, the CARS algorithm was employed to reduce the information dimension and select the spectral fingerprint features.

CARS is a frequent-used feature value extraction algorithm combining regression coefficients of PLS model and monte carlo sampling, which elements were similar with ‘natural selection’ [4, 18]. In this algorithm, adaptive reweighted sampling (ARS) selected the value with larger weight of regression coefficients in PLS model to build a fresh subcollection and dislodged the value with smaller weight. Then, the fresh subcollections were utilized to structure a PLS model. The spectral fingerprint features were defined as the wavelength which has the smallest root mean square error (RMSECV) in the PLS model after repetitive calculations [53].

#### Selection of significant vegetation indices

The number of wavelength points can be reduced quickly and availably and the accuracy of the model can be improved efficaciously by selecting significant VIs. ReliefF is a robust, successful and reliable attribute estimator, which can be able to provide the best weight vector and dispose noisy and flawed data [29]. ReliefF is widely used for selecting effective feature in the area of detecting plants diseases by VIs. Therefore, the ReliefF algorithm was used to single out the most sensitive VIs for training. In this study, 25 VIs were used to discriminate three conditions of strawberry leaves (healthy, gray mold and anthracnose). ReliefF makes use of the degree of distinction between the testing values and the near instances to calculate the weight of features [46]. Draw on their weights, the attribute of VIs will be arranged in rank by ReliefF. The vital procedure to highlight is that the 25 features weights should be initialized to zero at first.

### Development of the recognition model for strawberry disease

Hyperspectral imaging system intermixed with the chemometric methods, is recognized as a high efficiency, speedy, economical and practical, and nondestructive detection technology [40, 63]. In this study, BPNN, SVM and RF were chosen to develop the recognition models for strawberry diseases using different features. Spectral fingerprint features, VIs and the fusion features were considered as inputs to these three models, respectively. BPNN is a typical feed-forward learning algorithm, which consists of positive communication and signal error back-propagation, includes one input layer, one or more hidden layers and one output layer. [49]. There are large quantities of preponderances by using BPNN to classify the diseases, such as the architecture is much less complicated, the pattern is easy to build and the computation speed is fast which are conducive to the efficiency of disease classification [54]. SVM is an ideal measure to process data which is high dimension, nonlinear and noisy [30]. It takes advantage of structural risk minimization (SRM) principle to maximize the margin of class separation for better generalization performance of SVM [9]. It was proposed as the priority option for plant disease detection due to the promising performance. RF is a valid classifier which is capable of classifying data in high dimensions with many classes and acquire high precision [41, 52]. Plenty of independent classifiers (decision tree) construct the architecture of it. The voting results of each decision tree determines the class label of the input sample.

## Results

### Spectral behaviors

**Fig. 3:**
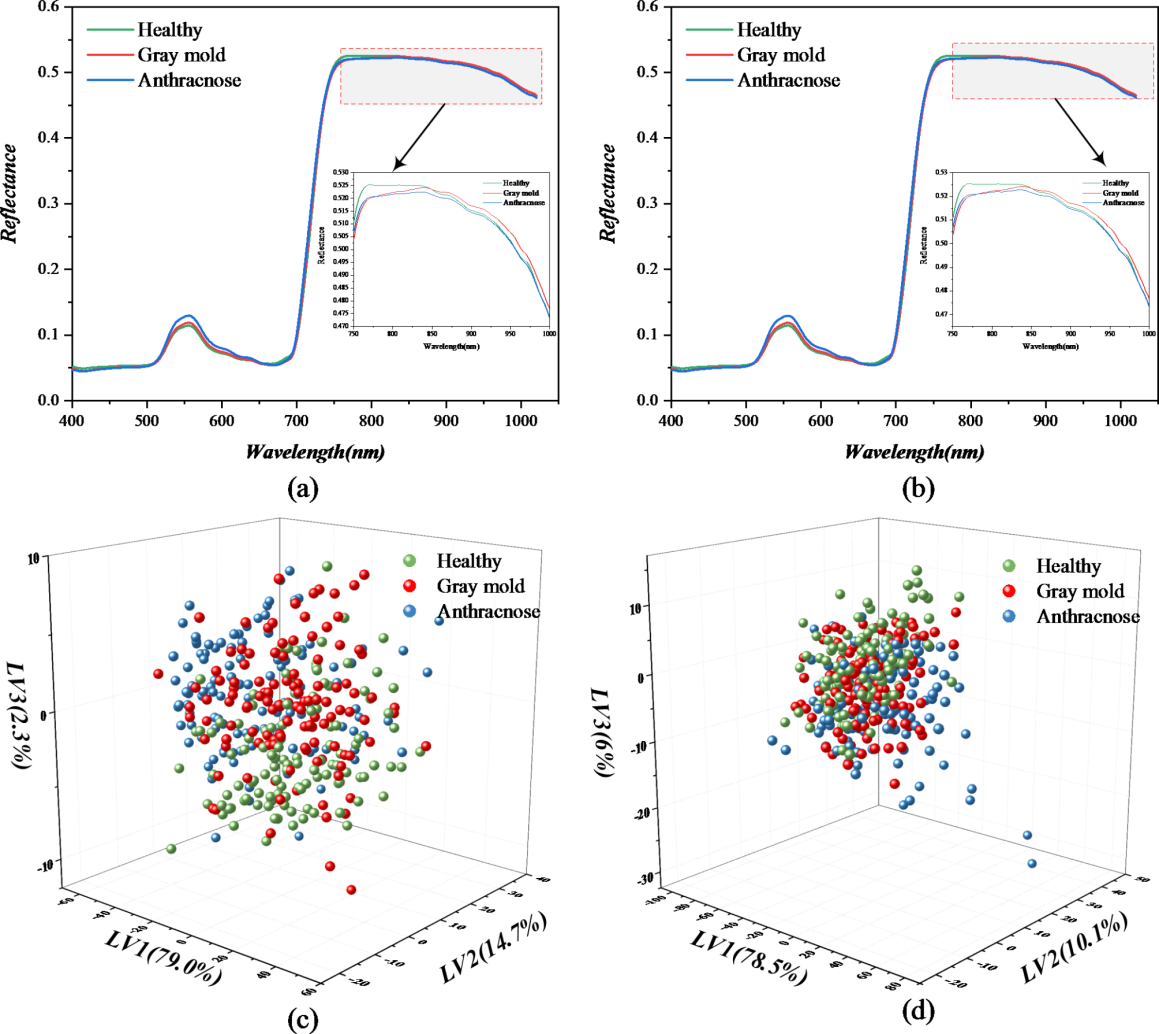
Result of the spectral reflectance curve and PCA of three types of leaves. (**a**) The original spectra. (**b**) The spectra after smooth process. (**c**) The PCA distribution of different types of leaves on original data. (**d**) The PCA distribution of different types of leaves on smooth data

The spectral reflectance curves and principal component analysis (PCA) distribution of three types of leaves were shown in the Fig. 3. It can be seen intuitively in Fig. 3 that there were no significant differences among the original spectral reflectance curves of healthy strawberry leaves and the leaves infected by gray mold and anthracnose disease. The three different types of spectral curve exhibited similar trends. In Fig. 2a, b, the spectrum (400–1000 nm) had a high absorption peak (550 nm) and a weak absorption valley (680 nm). The absorption peak at 550 nm was mainly the first overtone of O-H stretching and N-H stretching absorption in carbohydrates and proteins. The absorption valley at 680 nm was associated with the second overtone of CH and the stretching of CH2. In addition, a rapid increase in reflectance could be seen in the wavelength range of 680 to 750 nm, and from 750 nm to the end, the reflectance gradually decreased. In the wavelength range of 750–850 nm, the spectral reflectance of healthy strawberry leaves was higher than that of the suffered, and from 850 nm to the end, the reflectance of leaves with gray mold disease was higher than that of another two leaves. Figure 3c, d showed the diagrams of the three types of strawberry leaves using PCA, and it could be seen that the distribution of leaf samples of different categories was concentrated in the same region, which further indicated that similarity of these samples was relatively high. Therefore, it was difficult to detect diseases on strawberry leaves using spectral features alone.

### Vegetation indices analysis

**Fig. 4:**
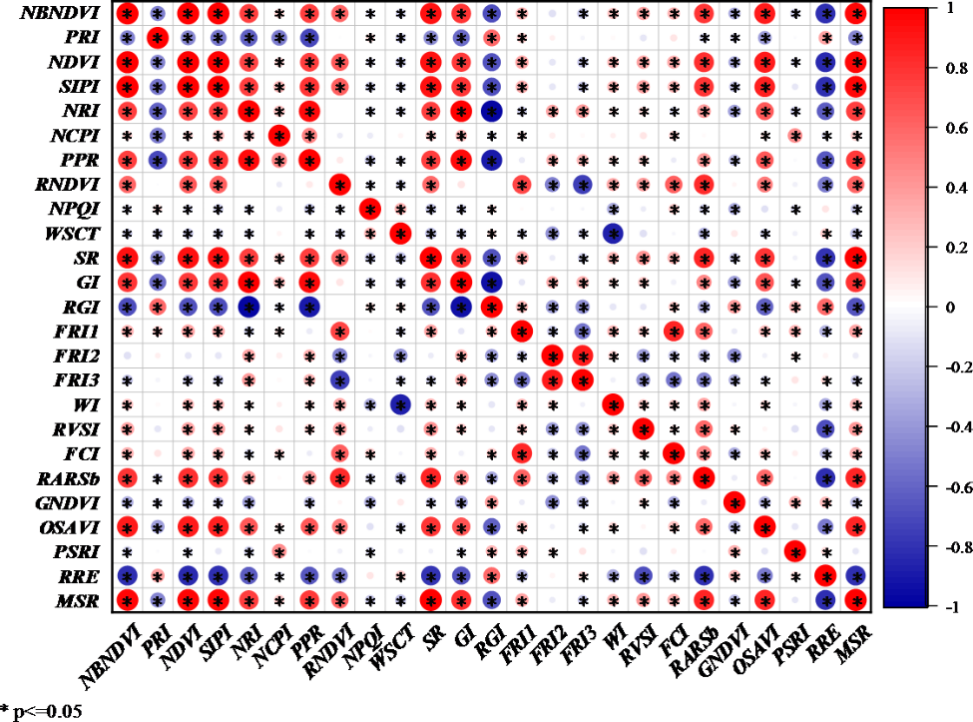
The correlation coefficients diagram of 25 vegetation indices

In this study, a total of 25 VIs was used for leaves diseases detection. Pearson correlation analysis is conducted in this study. The closer the absolute value of correlation coefficients between two different features to one, the stronger the correlation. The correlation coefficients between different VIs were presented in Fig. 4. Some indicators had strong correlations with others, but some do not. As an illustration, the PRI correlation index was quite high with the NBNDVI, NDVI, SIPI, NPI, NCPI, and PPR, (−0.53, −0.54, −0.62, −0.75, −0.63 and − 0.85, respectively) compared to RNDVI, FRI2, FRI3, WI, and PSRI (0, 0.14, 0, 0 and − 0.05, respectively) which had almost no connection with PRI. The FCI index and FRI1 index demonstrated high correlation coefficients (1.0). FRI3 had a strong correlation coefficient with RNDVI and FRI2 (0.9 and 1.0 respectively). Additionally, the NPQI, NCPI and GNDVI exhibited a phenomenon that was unconnected to any other index (0 ~ 0.2). The existence of correlation coefficient matrixes was of vital importance. The correlation analysis of different VIs offers a theoretical foundation for further VIs extraction and improved the robustness of the detection model.

**Fig. 5:**
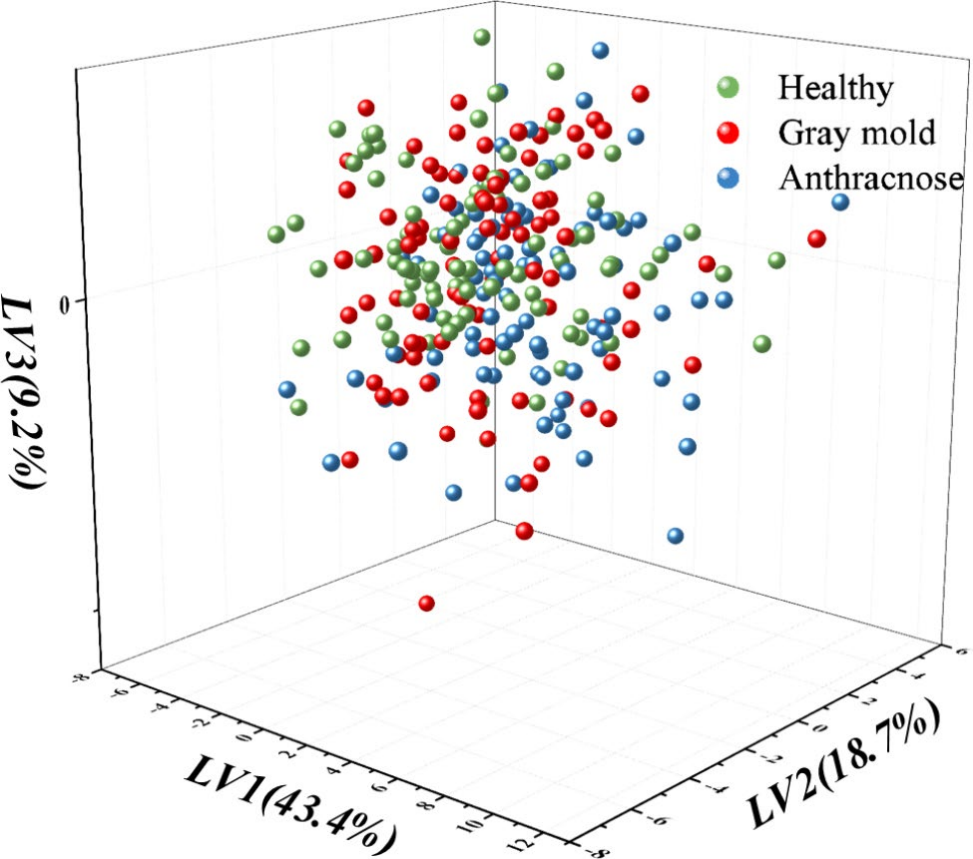
The PCA diagram of strawberry leaves based on vegetation indices

In addition, the PCA distribution of different types of leaf samples based on VIs was shown in Fig. 5. The figure revealed that the distribution of healthy, gray mold and anthracnose leaves in PCA was quite concentrated and mixed. As could be seen from the figure clearly, the green, red and blue particles representing healthy, gray mold and anthracnose leaves of strawberries were densely distributed and mixed. It also demonstrated that the VIs between healthy and infected leaves were similar. As a result, it was quite difficult to differentiate healthy, gray mold and anthracnose leaves using the VIs alone. Therefore, the experiment simultaneously combined spectral fingerprint features and VIs as inputs to achieve accurate detection of strawberry leaves diseases.

### Selection of spectral fingerprint and vegetation indices for strawberry disease detection

In this study, a total of 616 original spectral features and 25 VIs were used for strawberry diseases detection. In order to improve the performance of the diseases detection models and speed up the computation, this study respectively applied CARS and ReliefF algorithm to further extract the spectral fingerprint features and VIs.

Extraction of spectral features using CARS.

**Fig. 6:**
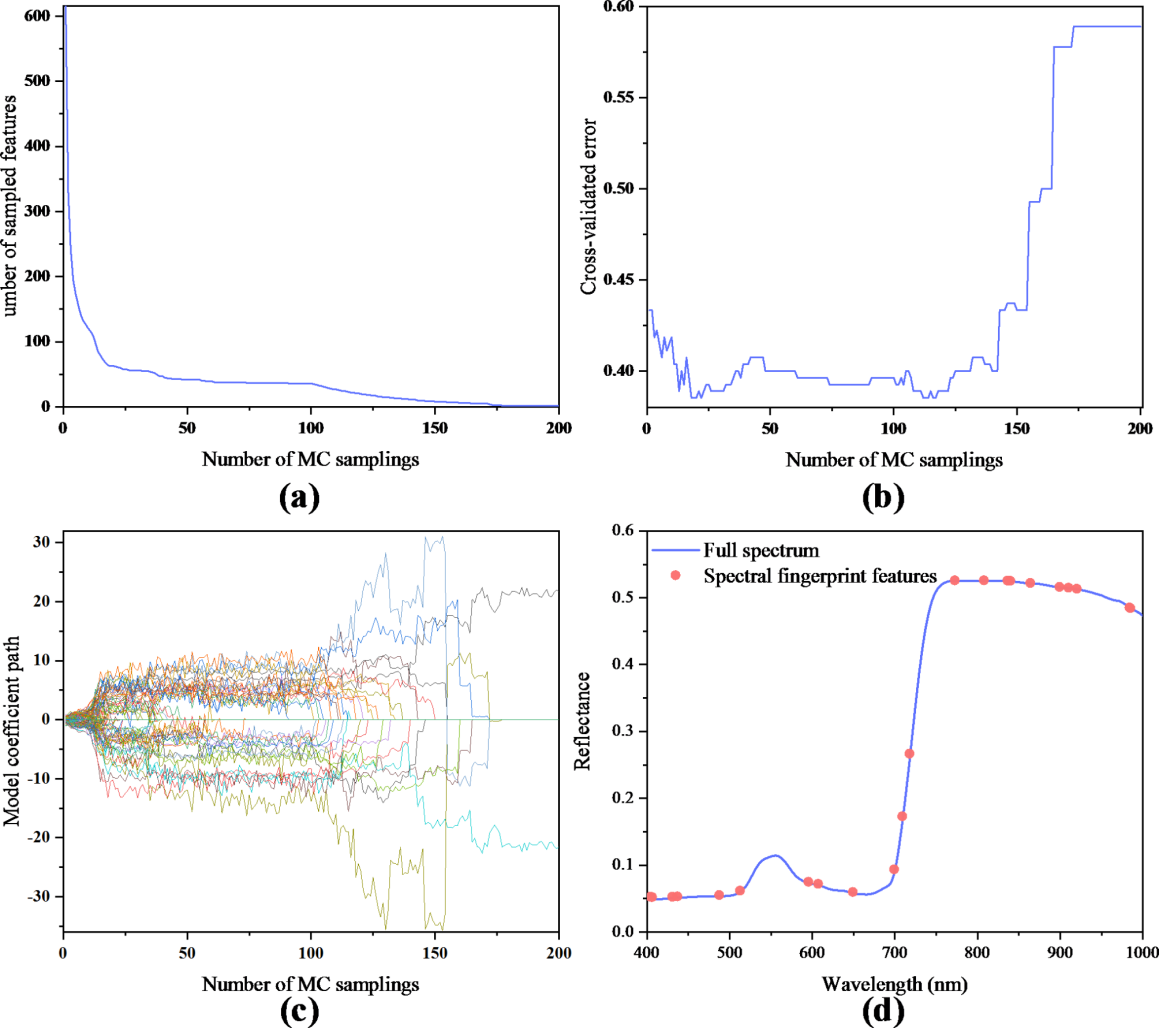
Result of extraction of the optimal features. (**a**) The number of sampled variables. (**b**) 10-fold RMSECV values. (**c**) regression coefficients of each variable. (**d**) Spectral fingerprint features distribution

During the running time of CARS algorithm, some irrelevant wavelengths were removed and some important features which contained more feature information were extracted. Figure 6a–c showed extraction of spectral fingerprint features. Finally, 22 optimal spectral fingerprint features were obtained in this experiment, and their distribution over the full spectral range was shown in Fig. 6d.

### Extraction of vegetation features using ReliefF

**Fig. 7:**
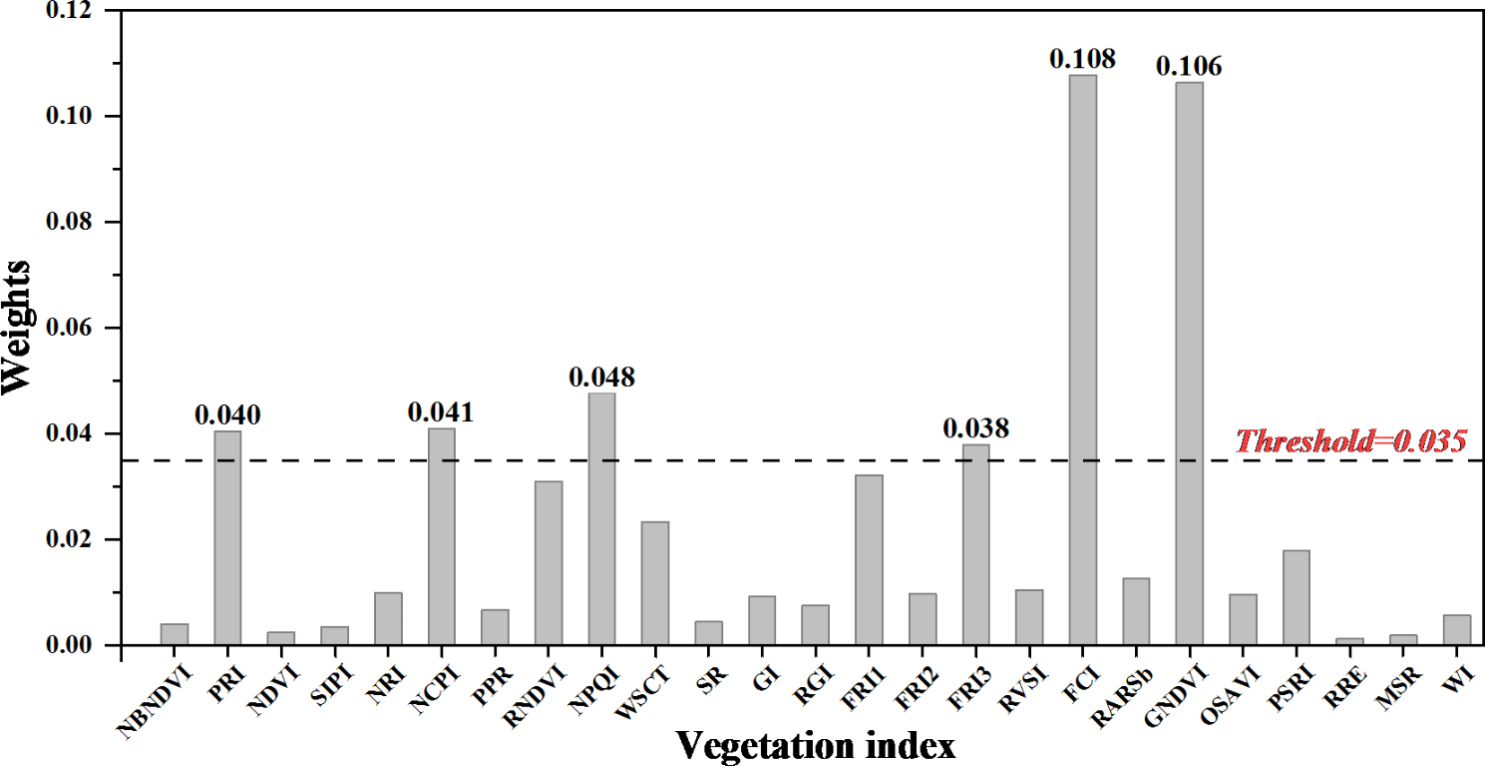
Result of weight coefficient analysis of 25 extracted features

**Fig. 8:**
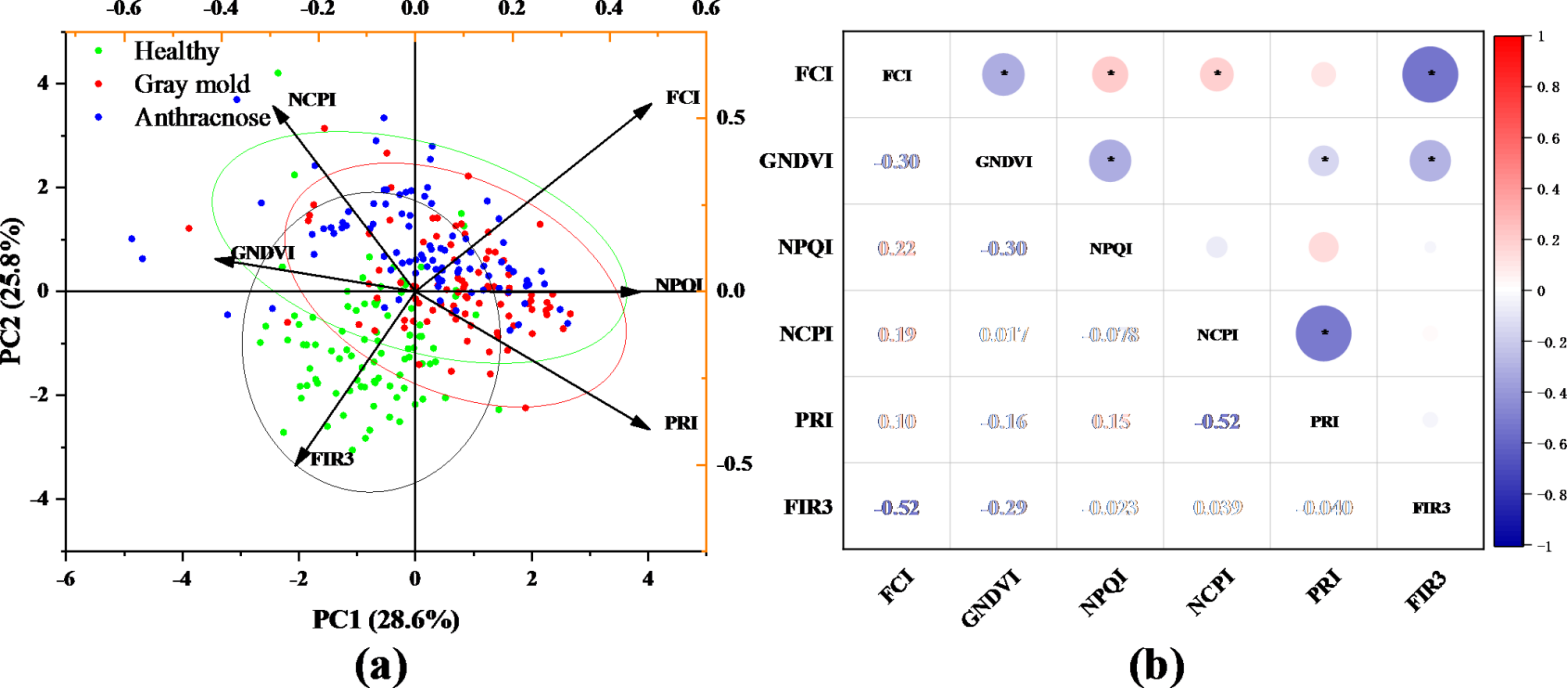
Result of Relief algorithm. (**a**) PCA of the six vegetation indices. (**b**) The correlation coefficients among the 6 vegetation indices

The ReliefF algorithm was used to sort the weights of 25 VIs. The result of weight coefficient analysis of 25 VIs was shown in Fig. 7. After repeated experiments, a threshold value of 0.035 was set and then the VIs with a weight greater than the threshold value were selected, and 6 VIs (PRI, NCPI, NPQI, FRI3, FCI, GNDVI) were taken eventually for subsequent classification. Among them, PRI was sensitive to changes in carotenoids in living plants, which can indicate photosynthetic light utilization and carbon uptake efficiency. NCPI was derived from reflectance at the visible light range to estimate the composition and abundance of plant pigments, and it was associated with photosynthetic efficiency and composition of stress related pigments. NPQI was identified as one of the most discriminative indices in early stages, in more details, the NPQI was more sensitive to the chlorophyll degradation into pheophytin. FRI3 and FCI both respond to leaf physiology. GNDVI was a more accurate measure of chlorophyll content than NDVI. Figure 8a showed the PCA distribution of healthy and infected leaves based on 6 VIs. The correlation coefficients among the 6 VIs are presented in the Fig. 8b. It can be seen from Fig. 8b that these 6 VIs were not strongly correlated with each other, the FCI correlation index was quite low with the GNDVI, NPQI, NCPI, PRI, FIR3 (−0.3, 0.22, 0.19, 0.3, −0.52, respectively). The GNDVI correlation index with NPQI, NCPI, PRI, FIR3 is −0.3, 0.0, −0.18, −0.29 respectively. The NPQI correlation index with NCPI, PRI, FIR3 is 0.0, 0.15, 0.0 respectively. The NCPI correlation index with PRI, FIR3 is −0.52 and 0.0. The correlation index between PRI and FIR3 is 0.0. The low correlation index indicated that they all have good independence.

### Comparison of the performance of models with different features

After feature extraction, spectral fingerprint features and VIs were combined as the inputs of the classifiers in this experiment. To compare the impact of different features on diseases detection, three different classifiers, BPNN, SVM, and RF, were developed in this experiment. The classification accuracies of diseases detection on strawberry leaf using various characteristics and classifiers were presented in Table 2. Table 2 showed that the fusion features, which combined spectral fingerprint features and VIs, had greater identification accuracy when compared to a single feature in three classifiers, and it also provided a greater improvement in the efficiency of the model. It was reasonable to believe that the combination of spectral fingerprint features and VIs was quite helpful to the early categorization of strawberry leaves diseases. Fusing the two features as inputs to machine learning models might be more effective in early perception. As a result, the model based on fusion features might obtain the greatest classification performance, which provided better robustness and classification accuracy. Among the detection models developed using fusion features, the BPNN classifier had the highest classification accuracy of 97.78%, followed by the SVM and the RF classifier, which had respective accuracy ratings of 94.44% and 93.33%. The reason for this phenomenon may be that there was a certain nonlinear relationship between fusion features and disease coefficients. It illustrated that the BPNN model had the ability to deliver higher accuracy and stronger generalization performance, which can work as an excellent model to spot early diseases in strawberry leaves. However, a BPNN classifier with a more complicated network structure required a longer operating time when the efficiencies of these methods were taken into account. But the amount of time needed was still within the acceptable range.

In addition, the spectral fingerprint features selected in the model only account for 3.57% of the original spectral data, and the VIs only occupy 24% of the original data, which greatly reduced the burden of the computer and improves the efficiency of the model. In actual operation, identification time and detection efficiency were of vital importance in detection factors. Therefore, the fusion of the two features was more conducive to the detection of strawberry leaves diseases, which can provide better detection accuracy and robustness.

**Table 2:** Classification accuracies of different input features and classifiers

Classifier	Spectral fingerprint features				Vegetation index features				Fused features			
	Healthy	Gray mold	Anthracnose	**Overall**	Healthy	Gray mold	Anthracnose	**Overall**	Healthy	Gray mold	Anthracnose	**Overall**
BPNN	100%	83.33%	66.67%	83.33%	86.67%	83.33%	73.33%	81.11%	100%	96.67%	96.67%	97.78%
SVM	93.33%	93.33%	56.67%	81.11%	93.33%	93.33%	76.67%	87.78%	100%	96.67%	86.67%	94.44%
RF	93.33%	70%	53.33%	72.22%	86.67.%	90%	76.67%	84.44%	100%	93.33%	86.67%	93.33%

## Conclusions

This study investigated the feasibility of applying hyperspectral imaging combined with spectral fingerprint features and VIs for early detection of gray mold and anthracnose on strawberry leaves. The characteristic difference between fungal infected and healthy strawberry leaves was very small in the early stage, which was difficult to be observed. In this study, 616 original spectral features and 25 VIs were used to detect strawberry diseases. In order to improve the performance of the disease detection model and speed up the computation, the CARS and ReliefF algorithm were used to further extract the spectral fingerprint features and VIs, respectively. Finally, a total of 22 optimal spectral fingerprint features and 6 important VIs (PRI, NCPI, NPQI, FRI3, FCI, GNDVI) were extracted. After that, three machine learning models, BPNN, SVM and RF, were developed for the early identification of strawberry gray mold and anthracnose, respectively, using spectral fingerprint, VIs and their combined features as inputs. The results showed that the combination of spectral fingerprint and VIs had better recognition accuracy compared with individual features as inputs, and the accuracies of the three classifiers were 97.78%, 94.44%, and 93.33% (BPNN, SVM and RF), respectively. This result indicated that the fusion features approach proposed in this study can effectively improve the early detection performance of strawberry leaves diseases. In future research, more researches will focus on: (1) exploring more effective leaf features for better feature fusion; (2) increasing the number of samples with different disease infestation levels to further validate the effectiveness and robustness of the algorithm; and (3) building deep learning frameworks to replace the traditional machine learning methods to detect early disease infection in strawberry leaves.

## Data Availability

The datasets used and/or analysed during the current study are available from the corresponding author on reasonable request.
